# Comparison of eri and tasar silk fibroin scaffolds for biomedical applications

**DOI:** 10.1007/s40204-016-0047-5

**Published:** 2016-03-08

**Authors:** Muthumanickkam Andiappan, Tinesh Kumari, Subramanian Sundaramoorthy, Gowri Meiyazhagan, Prasath Manoharan, Ganesh Venkataraman

**Affiliations:** 1 Department of Textile Technology, Anna University, Chennai, India; 2 Sri Ramachandra Medical University, Chennai, India

**Keywords:** Biomedical, Blood compatibility, Eri silk fibroin, Protein, Sterilization, Tasar silk fibroin, Tissue cell culture

## Abstract

The cultivated silk, mulberry, is being used as biomaterial in different forms. Eri, tasar and muga are some of the known wild silk varieties. The studies on biomedical applications of electrospun mats produced from these wild silks are limited though few studies on eri silk are available. In this work, comparison was made between eri and tasar silk fibroin scaffolds for biomedical application. The scaffolds were produced from eri silk fibroin (ESF) and tasar silk fibroin (TSF) by electrospinning method and they were treated with ethanol to improve dimensional stability. Ethanol treatment increased the crystallinity% of both ESF and TSF scaffolds. The crystallinity percentage of the ESF and TSF scaffolds was found to be 46.7 and 42.8 % respectively. Thermal stability was higher for ESF than that of TSF scaffold. The hemolytic % of ESF and TSF scaffolds was found to be 1.3 and 7.7 % respectively. The platelet adhesion on the surface of ESF scaffold was lower than that found on TSF scaffold. Better fibroblast cell attachment, binding and spreading was found on the ESF scaffold. The cell viability on ESF scaffold was 83.78 % and in TSF was 78.01 % for 48 h. The results showed that ESF electrospun scaffold can be considered as a better biomaterial for biomedical applications compared to that of TSF scaffold.

## Introduction

Silk is identified as one of the important biomaterial due to its biocompatibility, biodegradability and presence of natural proteinthough it is largely consumed for clothing applications. It is being used as suture material because of its higher tensile strengthand bio-resorbable properties. Silk can be extracted and decomposed easily by nature (Mei-po Ho et al. 2012 *)*. Silk consists of two components, fibroin (80 %) and sericin (20 %). Fibroin is a water insoluble protein with highly oriented crystalline structure and sericin is a gummy substance which is removed during degumming process; it is one of cause for inducing inflammatory reaction (Meinel et al. 2003; Santin et al. 1999). Silk is characterized by a highly repetitive primary sequence that leads to significant homogeneity in secondary structure, i.e., triple helices ß-sheets. These types of proteins usually exhibit impressive mechanical properties and hence provide an important set of material options in the field of controlled release and scaffolds for tissue engineering (Altman et al. 2003).

Silk is broadly classified as wild silk (Eri, Tasar, Muga) and domestic silk (mulberry). In the mulberry silk (Bombyxmori), glycine, alanine and serine constitute about 82 % of the amino acids, whereas, it is 73 % in the non-mulberry silks with a high proportion of alanine. The hydrophilic to hydrophobic amino acid ratio for non-mulberry silks (9.06–9.85) is higher compared to that of the mulberry varieties (5.29–6.22), which results in higher moisture content of non-mulberry silks (Altman et al. 2003; Sen and Murugesh Babu 2003). Silk fibroin has RGD (arginine-glycine-aspartic acid) sequence, which enhances cell adhesion, cell proliferation and differentiation (Muthumanickkam et al. 2013b; Bray et al. 2013). Silk I is the helical protein present in the silk glands prior to spinning. When the silk fibroin is treated with ethanol, the water soluble silkI undergoes structural change to water insoluble silkII protein, which is also an effective sterilization approach to silk fibroin scaffolds (Zhang et al. 2012). Eri silk fibroin (ESF) is composed of about 100 repetitions of alternating polyalanine (Ala^12−13^) and glycine-rich domains (Nakazawa et al. 2003). The glycine motifs are basically present in the random coil state structure, and it provides flexibility to the silk fibre, whereas the alanine rich motifs support to form crystalline ß-sheet structure. The sum of Gly and Ala residues in Eri silk is 82 % (Nakazawa et al. 2003; Huemmerich et al. 2006). The presence of sericin in Eri silk (*Samiacynthiaricini*) is less than that of mulberry silk, and also possesses higher amount of moisture regain than mulberry silk. ESF has higher content of hydrophilic and positively charged amino acids, which enhances the cell attachment and proliferation on the scaffold (Sen and MurugeshBabu 2004; Min et al. 2004).

The scaffolds fabricated from tropical Tasar (*A. Mylitta*) silk fibroin (TSF) has higher compressive strength over those made from other naturally derived materials such as collagen and chitosan (Mei-po Ho et al. 2012). The presence of the tripeptide sequence of Arg-Gly-Asp (RGD) in TSF acts as a biological recognition signal to promote cell adhesion, and consequently make TSF suitable for biomedical applications (Min et al. 2004). The higher alanine content in Tasar silk favors a distinct crystalline morphology; larger crystallites and lower crystallinity in non-mulberry varieties yield higher elongation %. Tasar shows the highest moisture regain value (10.76 %), followed by eri (10.21 %) and muga (9.82 %) for the outer layers. The higher moisture regain of non-mulberry silks suggests that all three non-mulberry silk varieties may consist of a higher ratio of hydrophilic to hydrophobic amino acid residues in their chemical architecture. Studies on application of mulberry silk for biomedical applications and comparison of eri and mulberry silk have been carried out by Muthumanickkam et al. (2013b). In the present work, a comparison was made between Eri silk (*Samiacynthia ricini*) and Tasar silk (*A. mylitta*) fibroin scaffolds. The scaffolds were produced by electrospinning method and subsequently treated with ethanol to improve dimensional stability. The physical and chemical characterization was carried out using Scanning electronic microscope (SEM), Thermogravimetric analyzer (TGA), FTIR spectrometer and X-ray diffractometer (XRD). The blood compatibility and platelet adhesion on the scaffold surface was examined. The fibroblast cells were used to evaluate the cell attachment and cell viability on the silk fibroin scaffolds.

## Materials and methods

### Preparation of scaffolds

Eri and Tasar silks were degummed using 0.5 M sodium carbonate and 1 g per litre of soap solution boiling at 95 °C under the pH range of 7.5–8.0 for removing sericin. The degummed silk (silk fibroin) was dissolved in Trifluroacetic acid (99.9 %) to prepare polymer solution. The fibroin solution was taken in a 2 ml syringe with the needle of inner diameter 1.3 mm and it was fixed on a infusion pump held in vertical position. The optimized concentration of 15 % (w/v) was maintained for Eri as well as Tasar fibroin solutions for producing nanofibrous scaffolds without any beads or spraying. The distance between the needle and the drum collector was kept at 15 cm, a 20 kV voltage was applied and the flow rate was set at 0.6 ml/h. In order to improve the dimensional stability, the scaffolds were immersed in ethanol for 1 h, which also sterilizes the material (Muthumanickkam et al. 2013b).

### Physical characterization of scaffolds

The eri and tasar silks were taken in various forms, viz., un-degummed silk, degummed silk, electrospun scaffolds without ethanol treatment and scaffolds with ethanol treatment. FTIR spectrometer (Perkin Elmer USA, PE 1600) in the region of 4000–500 cm^−1^ with 4 cm^−1^ resolution was used to analyze the change in functional groups in the above forms of silks. Scanning was carried out at a speed of 0.04 deg/s with a measurement range of 1°–70°. The area of scattering was measured using Fityk software. The X-ray diffract meter (Bruker USA, D8) with CuK-α radiation (*λ* = 1.54 A°) was used to determine the crystallite size and percentage of crystallinity of eri silk and tasar silk scaffolds; they were calculated using the Eqs. (1) and (2).

1 $$ {\text{Crystallite}}\,{\text{size}}\,\;({\text{A}}^{ \circ } ) = \frac{K\lambda }{\beta {\rm Cos}\theta } $$

Here, *k* is the shape factor, λ = 1.54 A°.

2 $$ {\text{Crystallinity}}\;(\% ) = \,\frac{{{\text{Total}}\;{\text{area}}\;{\text{of}}\;{\text{crystalline}}\;{\text{peak}}}}{{{\text{Total}}\,{\text{area}}\;{\text{of}}\;{\text{crystalline}}\;{\text{and}}\;{\text{amorphous}}\;{\text{region}}}} \times 100 $$

The thermal stability of the scaffolds was analyzed using the TGA (TA Instruments, Q500) at temperatures ranging from 37 to 700 °C in a nitrogen atmosphere at a heating rate of 20 °C/min.

The scaffold has to withstand the stress during cell culture and hence the tensile property of scaffold was tested under standard atmospheric condition using a Universal (Instron 3369) strength tester. The scaffold was cut into specimen of size 10 × 50 mm. The thickness of both the Eri silk and Tassar silk scaffolds was maintained at 0.16 mm. Glue tapes were fixed at the top and bottom of the scaffold and then it was clamped on the jaw of the tester; the gauge length was maintained at 30 mm and the test speed was kept at 20 mm/min.

Porosity is an important characteristic which would influence the tissue attachment and growth. The porosity of the scaffolds was measured by using a porosimeter based on capillary flow method. Scaffolds were cut into 5 × 5 × 1 mm pieces and the samples were impregnated with the wetting liquid. An inert gas N_2_ was used to displace this wetting liquid from the porous network. The pressure required to empty a pore corresponds to the pressure necessary to evacuate the liquid from the most constricted part of it. The Young–Laplace formula permits calculating of pore diameter from the measured pressure (Nazarov et al. 2004).

The water uptake of scaffold was measured as per the following method: Nanofibrous scaffold was dried in an oven at 60 °C under vacuum for overnight and the dry weight of scaffolds (*W*_d_) was measured. The scaffolds were immersed in distilled water at room temperature for 24 h. Then the excess water on the scaffold was removed using white tissue paper by applying uniform pressure, and the wet weight of the scaffolds (*W*_s_) was determined. The water uptake of scaffold was calculated using the Eq. 3.

3 $$ {\text{Water}}\;{\text{uptake}}\;(\% ) = \frac{{W_{\text{s}} - W_{\text{d}} }}{{W_{\text{d}} }} \times 100 $$

### Biological characterization

#### Hemolytic test

Blood compatibility of both ESF and TSF scaffolds was analyzed using hemolytic test. Human blood collected from a healthy volunteer in a 3.8 % sodium citrate coated tube was diluted with phosphate buffer saline (PBS) (pH 7.4) in the ratio of 1:20 (v/v). The blood diluted with PBS was taken as a negative control, and the blood with Triton X was taken as a positive control. The ESF and TSF scaffolds were treated with ethanol and then autoclaved. The scaffolds were immersed in 100 µL of blood and PBS solution followed by incubation at 37 °C for 60 min. Then, the samples were spun at 3000 rpm for 10 min. The optical density value (OD) of the supernatant was measured at 545 nm using spectrophotometer and the hemolytic rate was calculated using Eq. 4.

4 $$ {\text{Hemolytic }}\,(\% ) = \frac{{{\text{OD}}\;{\text{value}}\;{\text{of}}\,{\text{sample}} - \;{\text{OD}}\;{\text{value}}\;{\text{of}}\;{\text{negative}}}}{{{\text{OD}}\;{\text{value}}\;{\text{of}}\,{\text{positive}} - \;{\text{OD}}\;{\text{value}}\;{\text{of}}\;{\text{negative}}}} \times 100 $$

Platelet adhesion test was conducted to analyse the behavior of silk fibroin scaffolds while interacting with the human platelets. For this study, 5 mL of fresh human blood was collected from a healthy volunteer. The fresh blood was treated with 3.8 % sodium citrate, and spun at 3000 rpm for 10 min at 4 °C to obtain platelet-rich plasma (PRP). The platelet was placed on the scaffold and kept under incubation for 1 h. The platelet-attached ESF and TSF scaffolds were washed twice with PBS, and then immersed in PBS containing 2.5 % glutaraldehyde (pH 7.4) for overnight. They were subsequently dehydrated in gradient ethanol (20, 40, 60, 80, and 100 %) for 15 min and then dried in vacuum. The morphology of the platelets adhered on the scaffolds was characterized using SEM.

#### Cell culture

The scaffolds were treated with ethanol immediately after the removal of scaffold from the electrospinning machine to avoid curling and then it was autoclaved before the biological characterization. Rat L6 muscle fibroblasts were seeded at a density of 1 × 10^4^ cells per silk fibroin scaffold. The cells were incubated at 37 °C with 5 % CO_2_ for a period of 24 and 48 h. After the incubation, the scaffolds were removed from the well and rinsed with PBS twice to remove non-adhered cells from the scaffold. Then the scaffolds were fixed with 2.5 % phosphate-buffered glutaraldehyde and kept at 4 °C for 2 h and subsequently dehydrated with gradient ethanol solution (20, 40, 60, 80, and 100 %). The dried scaffolds were sputtered with iron and observed by SEM.

#### MTT assay

Rat L6 muscle fibroblasts were seeded at a density of 1 × 10^4^ per 96 well plates. After confluence, the scaffolds were placed on cells of the well plate. The cells treated with Triton X-100 were used as the positive control. After the requisite incubation time, 5 µL of MTT reagent (10 mg/mL) was added to the medium and incubated for 4 h at 37 °C, 95 % RH in an incubator containing 5 % CO_2_. Subsequently, the medium was discarded and 200 µL of dimethyl sulfoxide (DMSO) was added to record its optical density using spectrophotometer at 540 nm. The optical density value (OD) has varied linearly with the viable cell population. The cell viability percentage was calculated using Eq. 5.

5 $$ {\text{Cell}}\,{\text{viability}} = \frac{{({\text{OD}}\;{\text{value}}\,{\text{of}}\;{\text{sample}}\; ( {\text{treated}}\;{\text{well)} - \text{OD}}\,\;{\text{value}}\;{\text{of}}\;{\text{blank)}}}}{{({\text{OD}}\;{\text{value}}\,{\text{of}}\;{\text{control}}\; ( {\text{untreated}}\;{\text{well)} - \text{OD}}\,\;{\text{value}}\;{\text{of}}\;{\text{blank}})}} \times 100 $$

The statistical *t* test was conducted between the samples at 95 % confidence level.

## Results and discussion

### Physical characterization of scaffolds

#### SEM analysis

**Fig. 1:**
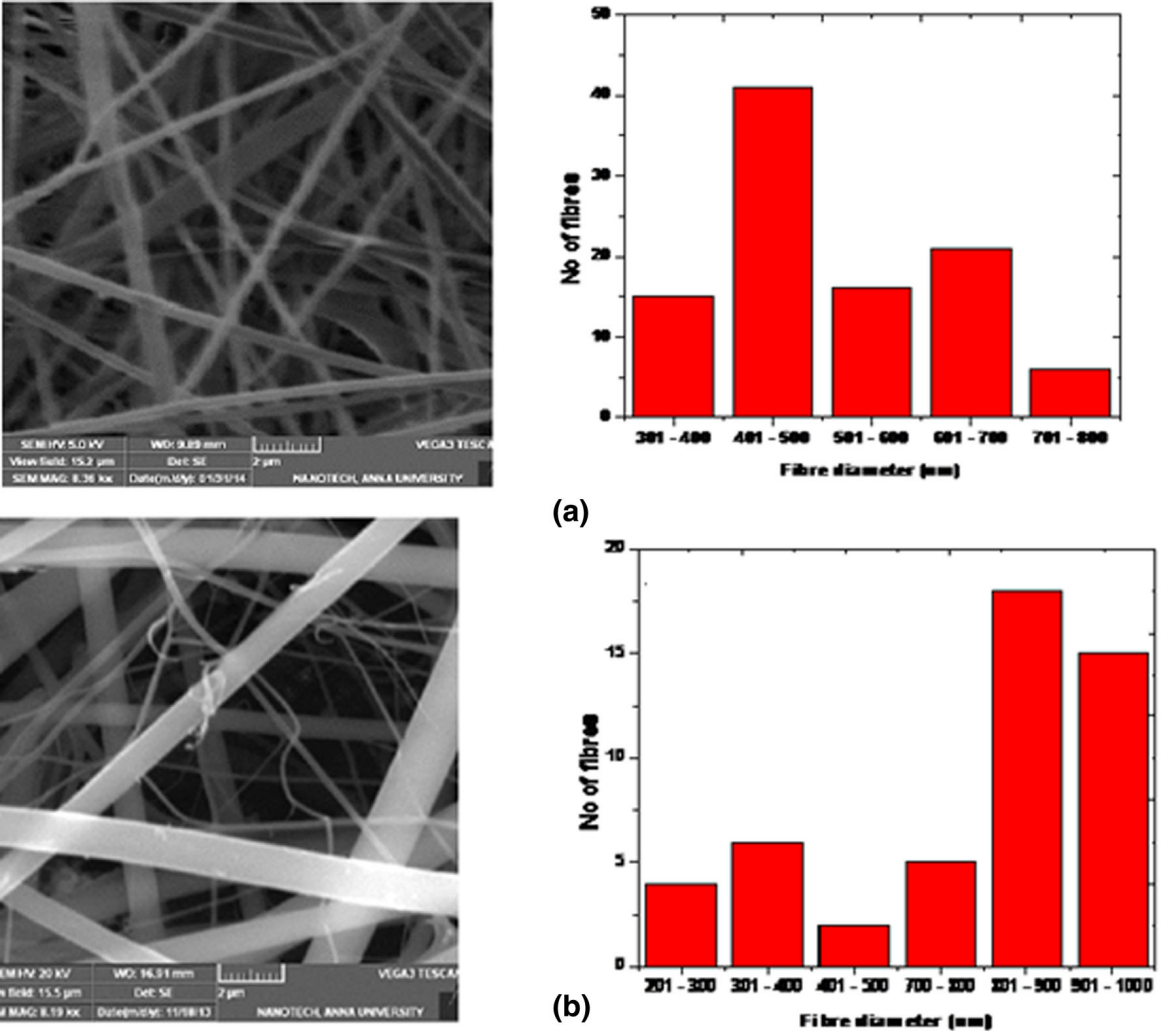
SEM image and histogram of **a** ESF and **b** TSF scaffolds

Figure 1a, b respectively show the SEM image and fibre diameter distribution of eri silk and tasar silk fibroin scaffolds without ethanol treatment. From the figures, it can be observed that majority of the fibres in ESF, TSF scaffolds have diameter in the range of 401–500 nm and 801–1000 nm respectively. The eri silk fibres have diameter lesser than the tasar fibres due to difference in molecular weight of fibroins of eri and tasar silks. The molecular weight of fibroin is associated with cystine content in the silk. The TSF has higher cystine content than ESF (Sen and MurugeshBabu 2004).

#### Thermal stability

**Fig. 2:**
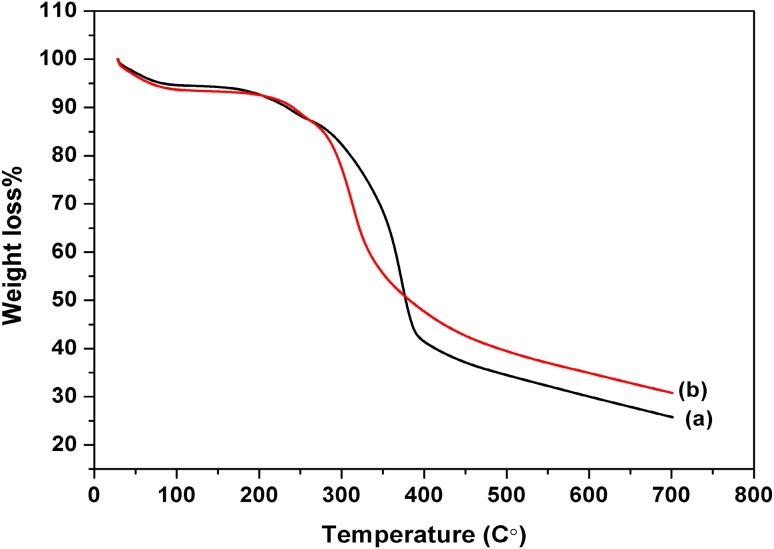
Thermogram of ethanol untreated *a* ESF and *b* TSF scaffolds

The thermogravimetric curves of ethanol untreated ESF and TSF scaffolds are shown in Fig. 2a, b respectively. The initial weight loss of the scaffolds occurs at around 100 °C due to the evaporation of water from the silk fibroin scaffolds. The second weight loss takes place at 380 and 350 °C respectively for eri silk and tasar silk.

**Fig. 3:**
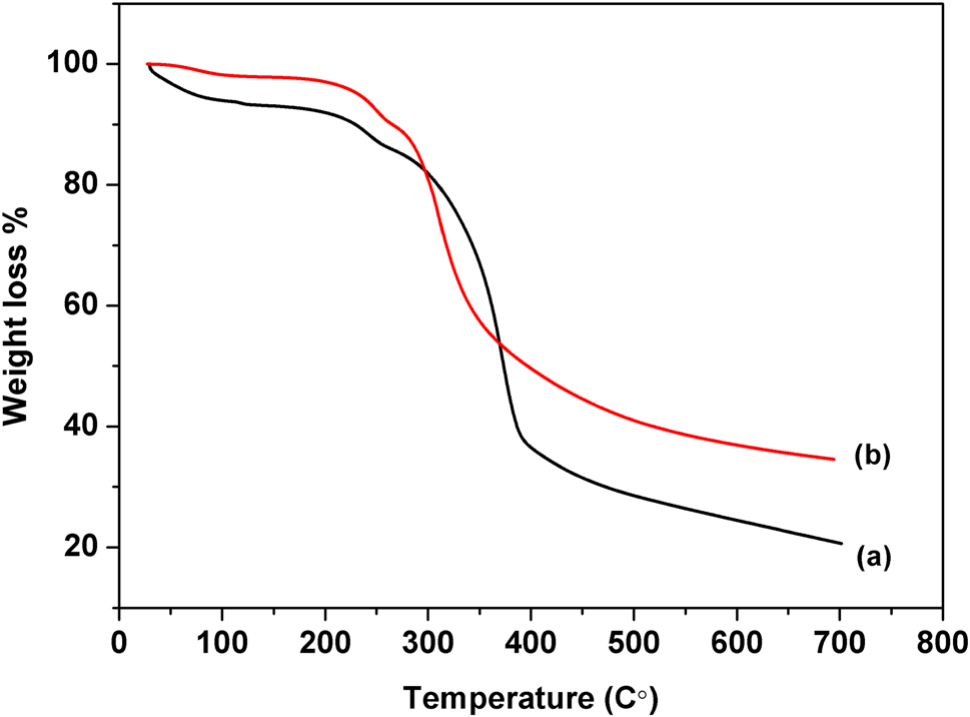
Thermogram of ethanol treated *a* ESF and *b* TSF scaffolds

The thermograms of ethanol treated ESF and TSF scaffolds are shown in Fig. 3a, b respectively. The initial weight loss of ethanol treated silk fibroin scaffolds occurs at around 100 °C due to the evaporation of water from the silk fibroin scaffolds. The second weight loss takes place at 390 and 370 °C and respectively for ESF and TSF scaffolds. The second weight loss of silk is due to breakdown of the side chain of amino group’s residuals as well as the cleavage of the peptide bond (Muthumanickkam et al. 2013b). The result shows that ESF scaffold has marginally better thermal stability than the TSF scaffolds in ethanol treated as well as untreated conditions.

#### FTIR analysis

**Fig. 4:**
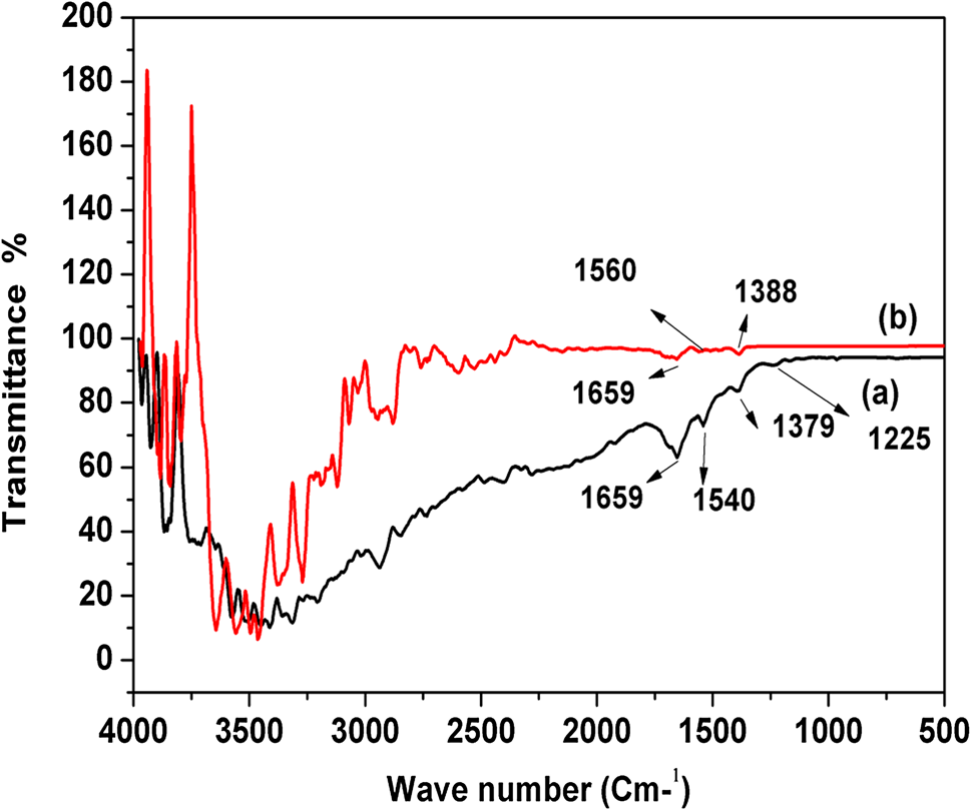
FTIR spectra of undegummed *a* eri and *b* tasar silk filaments

Figure 4a and brespectively shows the FTIR spectra of undegummed eri and tasar silks. The spectra shows the amide I absorption band at 1659 cm^−1^ (C=O stretching), amide II absorption band at 1540 cm^−1^ and 1560 cm^−1^ (N–H bending), and amide III absorption band at 1379 and 1388 cm^−1^ (C–N stretching) respectively for the undegummed eri and tasar silks. These absorption bands are attributed to the β-sheet structure of the silk fibroin (Simchuer et al. 2010; Muthumanickkam et al. 2010; NasimAmiraliyan and Kish 2009).

**Fig. 5:**
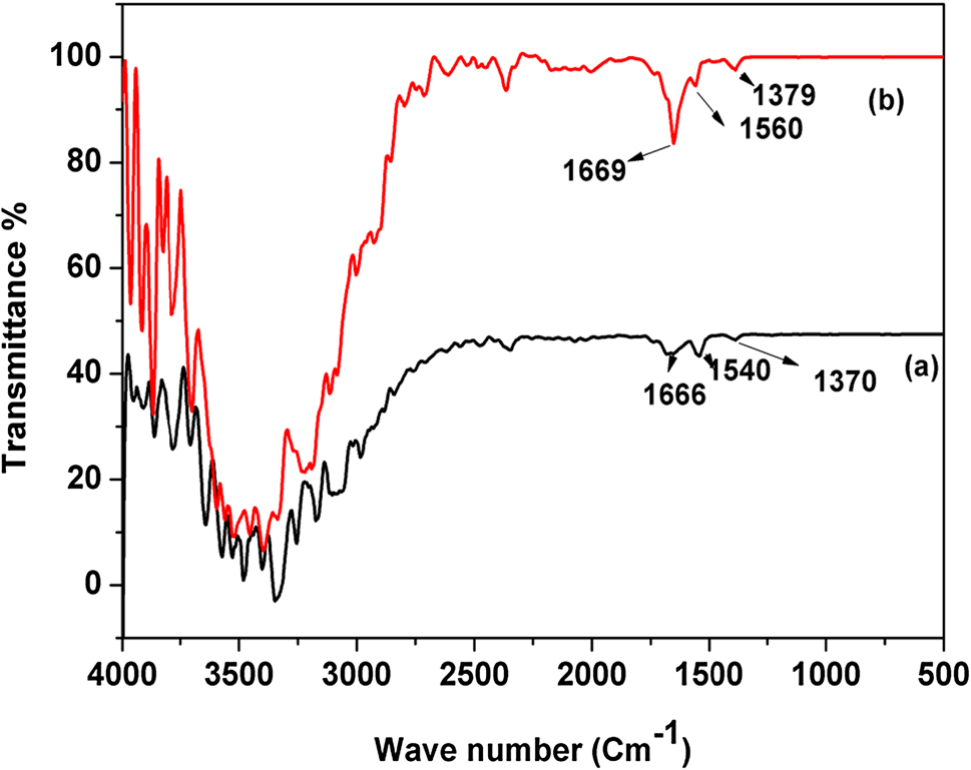
FTIR spectra of degummed *a* eri and *b* tasar silk filaments

Figure 5a, b respectively shows the FTIR spectra of degummed eri and tasar silks. The spectra shows the amide I absorption band at 1666 and 1669 cm^−1^, amide II absorption band at 1540 and 1560 cm^−1^, and amide III absorption band at 1370 and 1379 cm^−1^ respectively for the degummed Eri and Tasar silks. From the spectra (Figs. 4a, 5a), it could be observed that the wave number of amide I of Eri and Tasar silk has shifted from 1659 to 1666 cm^−1^ and 1659 to 1669 cm^−1^ respectively due to the degumming process. This may be attributed to the change from β-sheet structure to α-helix structure of silk due to degumming process (Muthumanickkam et al. 2013b).

**Fig. 6:**
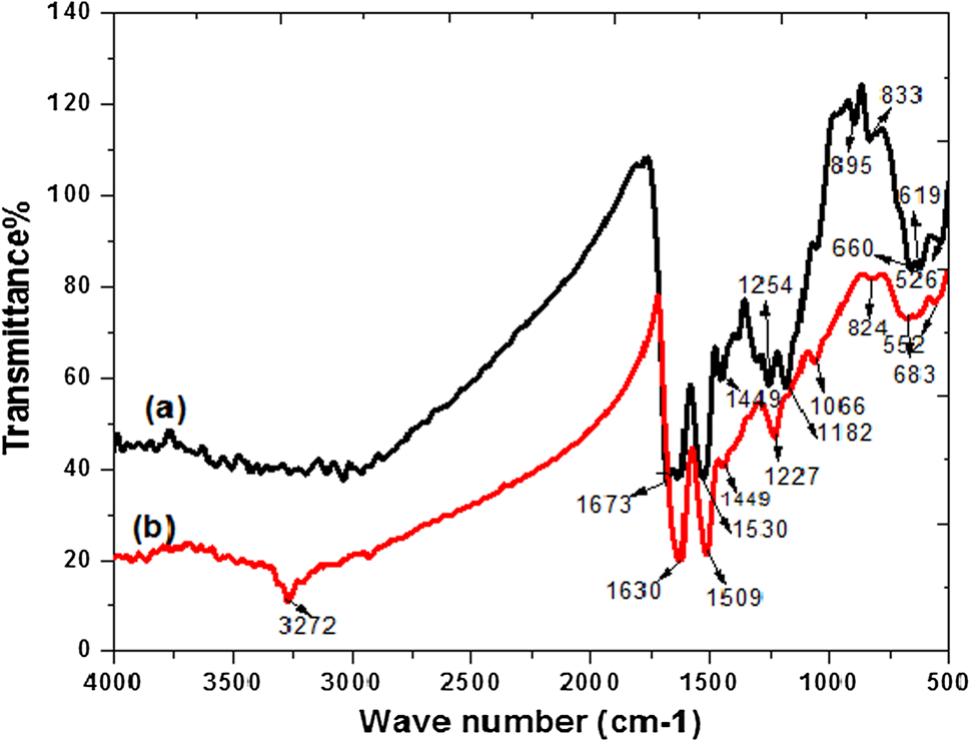
FTIR spectra of ethanol untreated *a* ESF and *b* TSF scaffolds

Figures 6a, b, respectively shows the FTIR spectra of ESF and TSF scaffolds without ethanol treatment. The spectrum (6a) shows the amide I absorption band at 1673 cm^−1^, amide II absorption band at 1530 cm^−1^ and amide III absorption band at 1254 cm^−1^for ethanol untreated ESF scaffold. The spectrum (6b) shows the amide I absorption band at 1630 cm^−1^ amide II absorption band at 1509 cm^−1^ and amide III absorption band at 1227 cm^−1^ for the ethanol untreated TSF scaffold.

**Fig. 7:**
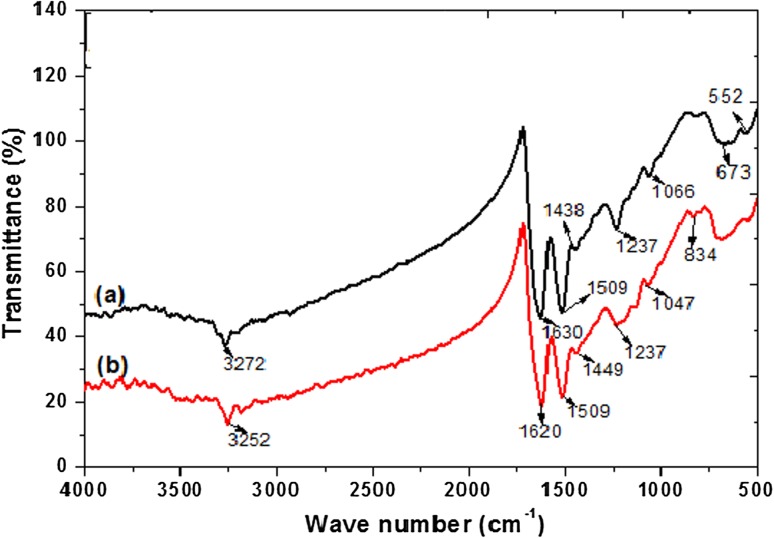
FTIR spectra of ethanol treated *a* ESF and *b* TSF scaffolds

Figures 7a, b, shows the FTIR spectra of ethanol treated ESF and TSF scaffolds. ESF scaffold (7a) shows the amide I absorption band at 1630 cm^−1^, amide II absorption band at 1509 cm^−1^ and amide III absorption band at 1237 cm^−1^. TSF scaffold (7b) showsthe amide I absorption band at 1620 cm^−1^, amide II absorption band at 1509 cm^−1^ and amide III absorption band at 1237 cm^−1^. The amide I absorption band has shifted from 1630 to 1620 cm^−1^ due to ethanol treatment of TSF scaffold, whereas the shift is from 1673 to 1630 cm^−1^ for the ethanol treatment of ESF scaffold. This shift may be due to change from α-helix to β-structure of the silkfibroin due to ethanol treatment. The ethanol treatment causes rearrangement of the hydrogen bonds in the silk fibroin nanofibrous scaffolds.

#### XRD analysis

**Fig. 8:**
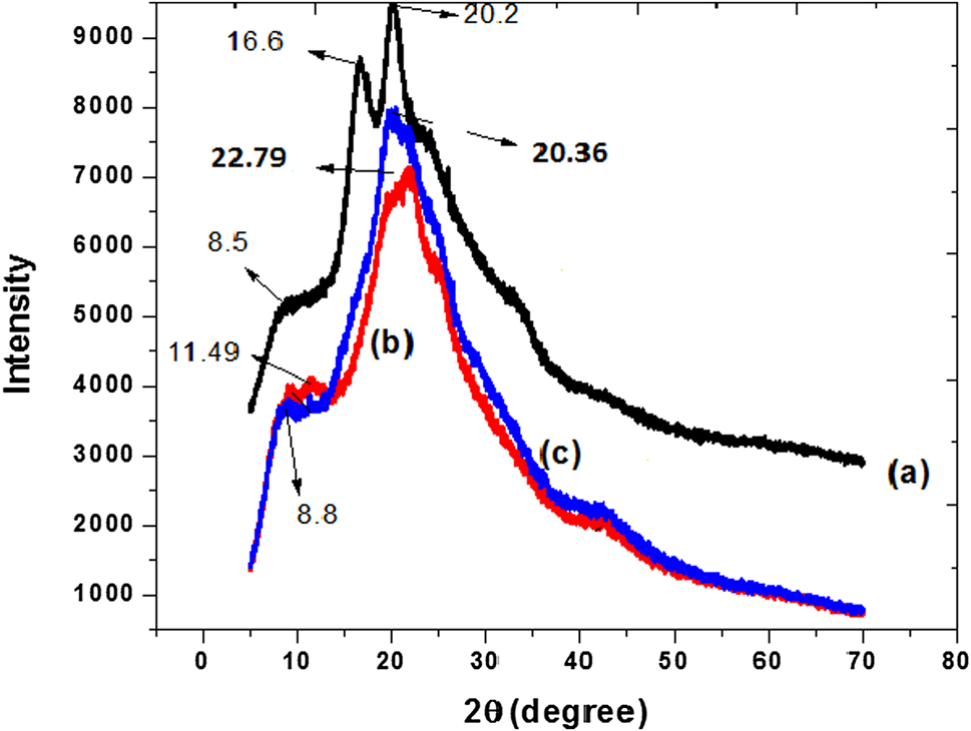
XRD diffrograms of *a* Undegummed Eri silk filament *b* Ethanol untreated ESF scaffold and *c* Ethanol treated ESF scaffold

Figure 8a, shows the diffraction peaks at 20.2°, 16.6° and 8.5° for undegummed eri silk, and their corresponding spaces are 4.39 A°, 5.34 A° and 10.4 A° respectively. The strong intensity peaks appearing at 20.2° and 16.6° may be attributed to the crystalline structure and the weak intensity peak appearing at 8.5° is due to the non-crystalline structure.

Figure 8b, shows the diffraction peaks at 22.79°, 11.49° and 8.8° for ethanol untreated ESF scaffold, and their corresponding spaces are 3.90 A°, 7.7A° and 10.04 A° respectively. The weak intensity peak appearing at 11.49° indicates amorphous content in the untreated ESF scaffold. Figure 8c, shows the diffraction peaks at 20.36° and 8.8° for ethanol treated ESF scaffolds and their corresponding spaces are 4.36 A° and 10.04 A° respectively. The intensity of the peak at 20.36° (Fig. 8c) is higher than the intensity of peak at 22.79° (Fig. 8b), which may be due to the conversion of part of the random coil structure to β-crystalline structure by ethanol treatment.

**Fig. 9:**
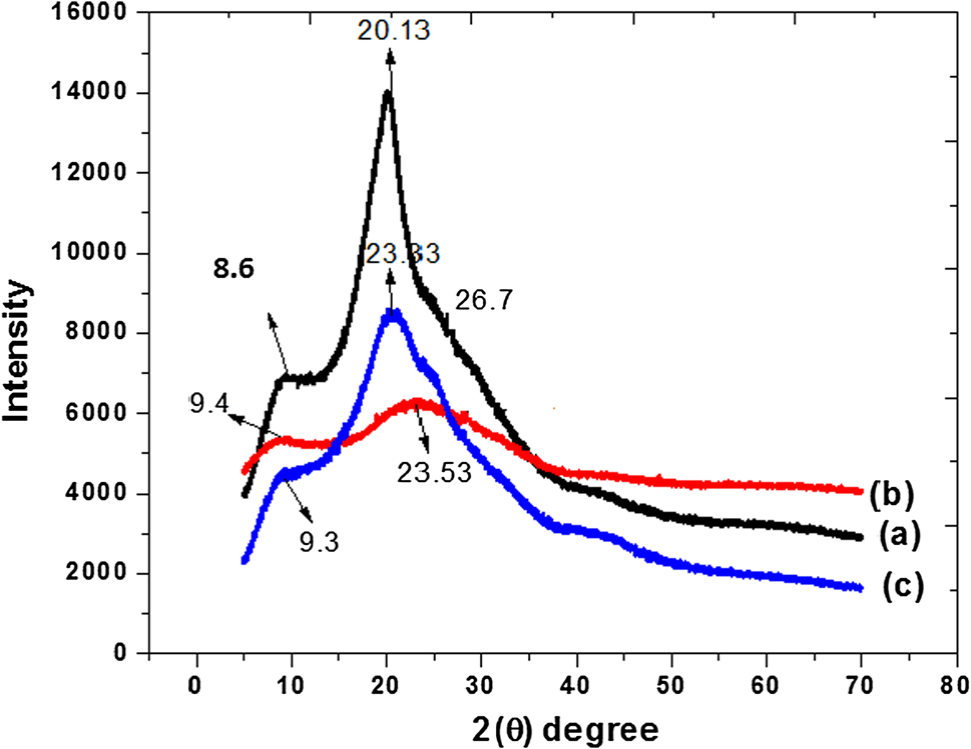
XRD diffrograms of *a* Undegummed Tasar silk filament *b* Ethanol untreated TSF scaffold and *c* Ethanol treated TSF scaffold

Figure 9a, shows the diffraction peaks at 20.13° and 8.6° for undegummed tasar silk and their corresponding spaces are 4.41 A° and 10.27 A° respectively. The strong intensity peak at 20.13° is due to the crystalline region. Figure 9b, shows the diffraction peaks at 23.53° and 9.4° for TSF scaffold without ethanol treatment and their corresponding spaces are 3.78 A° and 9.4 A°. Figure 9c, shows the diffraction peaks at 23.33° and 9.3° for ethanol treated TSF scaffold and their corresponding spaces are 3.81 A° and 9.5 A°. The intensity of the peak at 23.33° (Fig. 9c) is higher than the intensity of peak at 23.53° (Fig. 9b), which may be attributed to the conversion of part of the random coil structure to β-crystalline structure by ethanol treatment (Nazarov et al. 2004).

Table 1 shows the average crystal size and the crystalline percentage of eri and tasar silks, and ESF and TSF scaffolds.

**Table 1:** Crystallinity and crystallite size of eri and tasar silks and their scaffolds

Materials	Crystallinity (%)	Crystallite size (A°)
Undegummed eri silk filament	47.78	36.3
Ethanol untreated ESF scaffold	40.90	26.6
Ethanol treated ESF scaffold	46.73	33.3
Undegummed tasar silk filament	44.31	38.5
Ethanol untreated TSF scaffold	41.11	26.0
Ethanol treated TSF scaffold	42.77	38.5

Silk II structure (β-sheet structure) is connected with crystalline region and the silk I structure (random coil) structure is connected with amorphous region. The results show that crystalline percentage is higher in ethanol treated electrospun scaffolds than the untreated scaffolds. After the ethanol treatment, silk I structure changes into silk II structure. Ethanol treated ESF scaffold shows higher crystalline percentage than the TSF scaffolds due to higher alanine content present in the eri silk.

#### Porosity

**Fig. 10:**
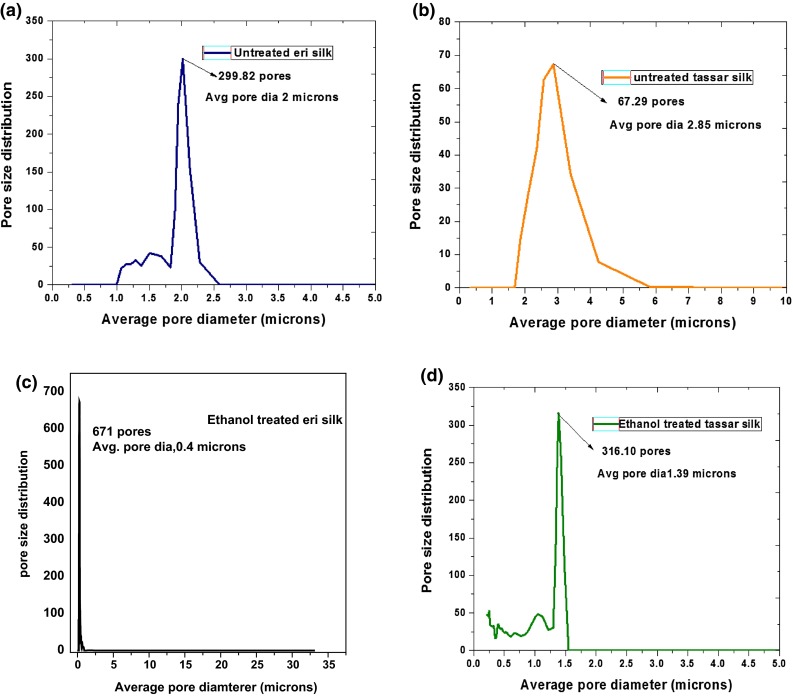
Pore size distribution of **a** Ethanol untreated ESF, **b** Ethanol untreated TSF, **c** Ethanol treated ESF and **d** ethanol treated TSF scaffolds

Figure 10a–d, shows the pore size distribution of ESF and TSF scaffolds with and without ethanol treatment. ESF scaffold without ethanol treatment in Fig. 10a shows that majority of the pores have diameter in the range of 1.5–2.3 μm with the mean pore diameter of 2.0 μm, whereas, the TSF scaffold without ethanol treatment (Fig. 10b) shows that majority of the poreshave the diameter in the range of 2–4 μm with the mean pore diameter of 2.85 μm.

Figure 10c, d, shows that majority of the pores have diameter in the range of 0.1–2 μm and 1.3–1.5 μm, with mean pore diameter of 0.4 and 1.39 μm respectively for the ethanol treated ESF and TSF scaffolds. When the electrospun mat is immersed in ethanol, it swells and shrinks (Muthumanickkam et al. 2010; NasimAmiraliyan and Kish 2009). The relative shrinkage of nanofibrous scaffolds after treatment leads to decrease in pore diameter and porosity (Thompson et al. 2007). The result shows that ESF scaffold has less range of pore size distribution and has less pore diameter compared to that of TSF scaffold due to the reason that the diameter of ESF fibres are less compared to that of TSF fibres. This may help for better cell attachment and proliferation on the scaffolds.

#### Water uptake

Table 2 shows the water uptake of ESF and TSF scaffolds with and without ethanol treatment. The water uptake percentage of ESF scaffold is lower than that of TSF scaffold due to higher hydrophilic to hydrophobic amino acids ratio in TSF scaffolds. It can be seen from the table that the water uptake reduces due to ethanol treatment. The reduction in water uptake is due to increase in crystallinity and reduction in pore size.

**Table 2:** Water uptake of ESF and TSF scaffolds

Materials	Water uptake (%)
Ethanol untreated TSF scaffold	75.34
Ethanol untreated ESF scaffold	73.54
Ethanol treated TSF scaffold	59.48
Ethanol treated ESF scaffold	57.18

#### Tensile strength

The mean tensile stress and strain values are 0.860 MPa, 4.87 % and 0.501 MPa, 3.0 % for untreated eri and tasar silk fibroin scaffolds respectively. The eri silk has higher tensile stress and tensile strain than that of tasar silk fibroin scaffold. The mean tensile stress and strain values are 1.375 MPa, 2.352 % and 1.414 MPa, 3.7 % for ethanol treated tasar and eri silk fibroin scaffolds respectively. From the results, it can be seen that the tensile strength is higher in eri scaffold than the tasar scaffold due to the presence of higher crystalline region in eri fibroin. As the result of ethanol treatment, the scaffold shrinks, which increases the fibre to fibre friction and cohesive force between the fibres in the scaffold, which in turn increases the tensile strength (Muthumanickkam et al. 2013a).

### Biological characterization of scaffolds

#### Hemolysis %

**Fig. 11:**
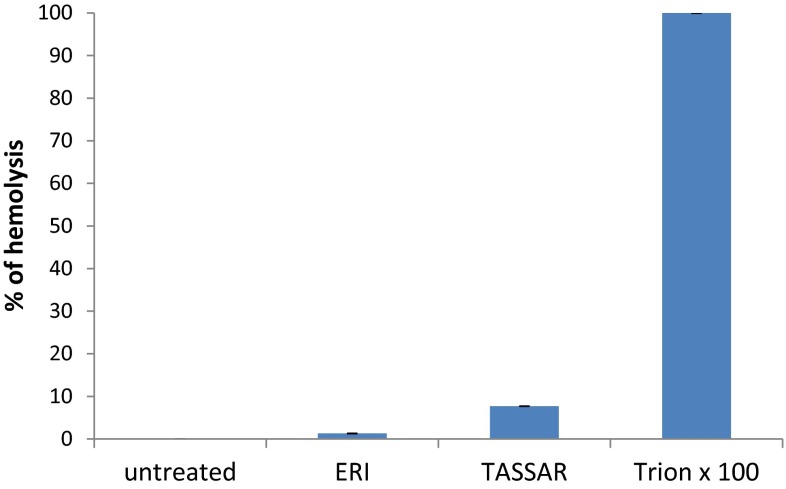
Hemolytic percentages of ESF and TSF scaffolds

The hemolytic percentage should be less than 5 % in order to use the material for biomedical applications. Figure 11 shows that the hemolytic percentage of ESF and TSF scaffolds is 1.3 and 7.7 respectively, which indicates that ESF has better blood compatibility and can be used as biomaterial.

#### Platelet adhesion

**Fig. 12:**
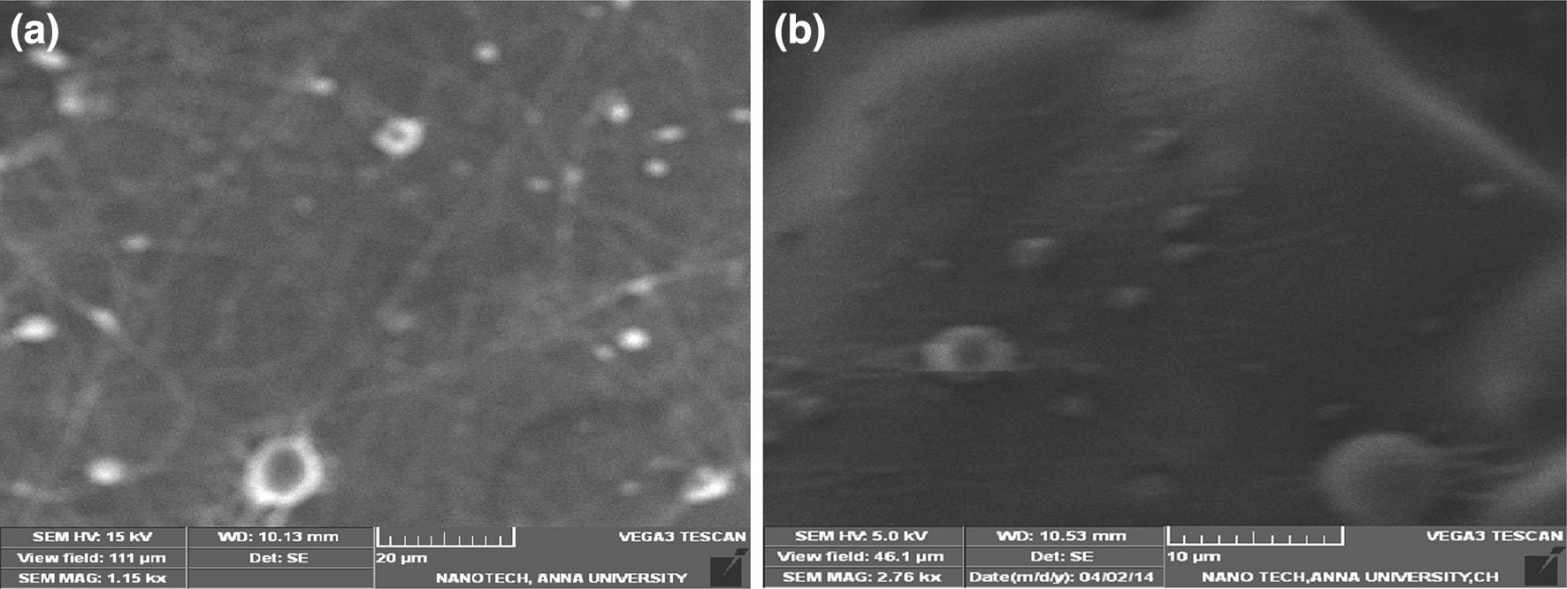
Platelet adhesion on **a** ESF and **b** TSF scaffolds

SEM images in Fig. 12a, b respectively, shows the platelet adhesion on the surface of ESF and TSF scaffolds. From the SEM images, it can be observed that ESF shows less platelet adhesion on the surface than the TSF scaffolds due to less hydrophilic ratio of eri silk (0.36) compared to that of tasar silk (0.44) (Huemmerich et al. 2006). The platelet non-adherence is important for the scaffolds to be used for biomedical applications.

#### Cell culture

The scaffolds are required to support cell attachment, growth, proliferation and maintain normal state of cell differentiation. To evaluate the initial cell attachment and spreading, the fibroblast L6 cells were seeded on the ESF and TSF scaffolds.

**Fig. 13:**
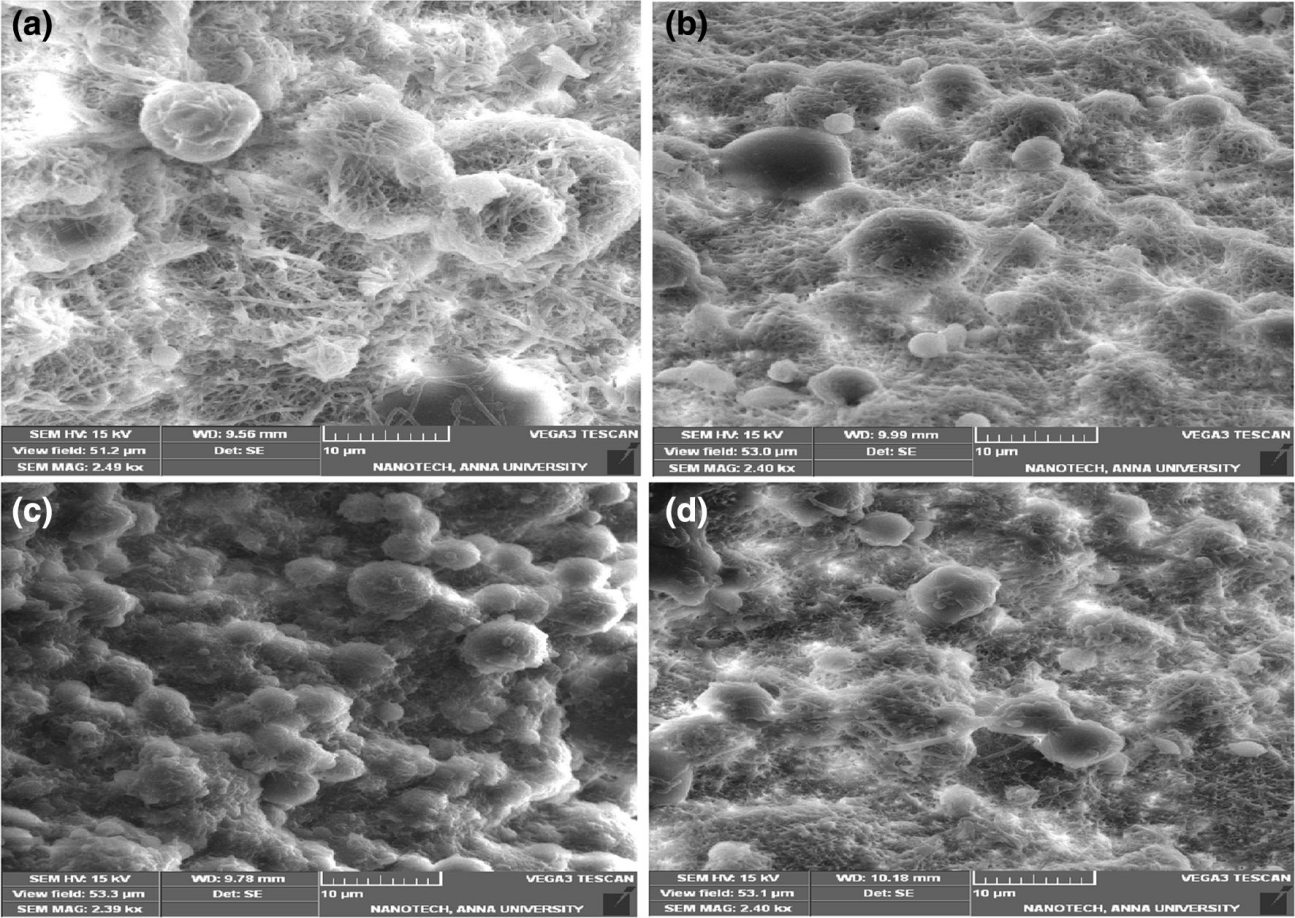
Fibroblast attachment on **a** ESF and **b** TSF scaffolds after 24 h and **c** ESF, **d** TSF scaffolds after 48

**Fig. 14:**
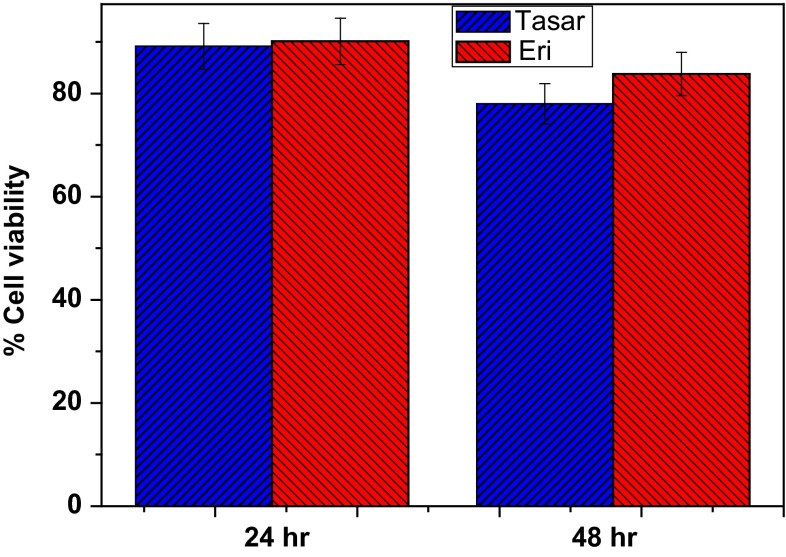
Cell viability of ESF and TSF scaffolds

SEM images in Fig. 13a, b, shows the fibroblast cell attachment and spreading on ESF and TSF scaffolds after 24 h and Fig. 14c, d, shows the cell attachment after 48 h on ESF and TSF scaffolds. It can be seen from the Fig. 13a, b that both ESF and TSF scaffolds show better L6 fibroblast cell attachment in 24 h of incubation. But after 48 h, TSF shows lesser cell attachment than the ESF. The cell attachment and spreading is higher in ESF scaffold due to higher amount of positively charged amino acids (Muthumanickkam et al. 2013a; Minoura et al. 1995; Patra and Talukdar 2012).

#### Cell viability

Figure 14, shows the cell viability of ESF and TSF scaffolds for the period of 24 and 48 h respectively. The percentage of cell viability in ESF and TSF scaffolds are 90.11 and 89.15 % for 24 h and 83.78 and 78.01 % for 48 h.

ESF shows marginally higher cell viability percentage than TSF scaffolds, however, statistically not significant.

## Conclusions

The eri silk and tasar silk scaffolds were produced by electro spinning method. The scaffolds were treated with ethanol to increase dimensional stability. Ethanol treatment increased the crystallinity percentage of both ESF and TSF scaffolds. Thermal stability of the ESF scaffold was found to be better than that of the TSF scaffold. The hemolytic percentage of TSF and ESF scaffold was found to be 7.7 and 1.3 % respectively, which indicates that ESF has better blood compatibility than TSF scaffold. The platelet adhesion on the ESF scaffold was less than the TSF scaffold. The cell attachment, binding and spreading on the ESF scaffold was superior compared to the TSF scaffold. In most of the characteristics, the ESF scaffold shows better performance compared to that of TSF scaffold and hence ESF can be considered a suitable biomaterial for biomedical applications.
